# Quantifying net loss of global mangrove carbon stocks from 20 years of land cover change

**DOI:** 10.1038/s41467-020-18118-z

**Published:** 2020-08-26

**Authors:** Daniel R. Richards, Benjamin S. Thompson, Lahiru Wijedasa

**Affiliations:** 1 Singapore-ETH Centre, ETH Zurich, Singapore, Singapore; 2 School of Social Sciences, Monash University, Melbourne, Australia; 3 Sydney Southeast Asia Centre, University of Sydney, Sydney, Australia; 4 ConservationLinks Pvt Ltd, Singapore, Singapore; 5 Integrated Tropical Peat Research Program, NUS Environmental Research Institute, National University of Singapore, Singapore, Singapore

**Keywords:** Carbon cycle, Tropical ecology, Environmental sciences

## Abstract

Mangrove forests hold some of the highest densities of carbon recorded in any ecosystem, but have experienced widespread deforestation through conversion to aquaculture and agriculture. Alongside deforestation, mangroves have shown simultaneous natural expansion in some parts of the world, and considerable investments have been made into restoration programmes. Here we estimate net changes in the global mangrove carbon stock due to land cover change between 1996 and 2016, using data on mangrove deforestation and forestation, and proportional changes in carbon stock during processes of mangrove loss and gain. The global mangrove carbon stock declined by 158.4 Mt (95% CI = −156.8–525.9 Mt); a reduction of 1.8% of the stock present in 1996. Efforts to conserve and restore mangroves appear to have had some success, and - along with natural forestation - have contributed to relatively low net losses of mangrove carbon stocks over two decades.

## Abstract (web summary)

Mangroves and the carbon they store are threatened by deforestation, but the efficacy of policies to protect them is unknown. Here the authors assess changes in mangrove carbon stocks between 1996 and 2016 and show less loss than previous methods estimated, indicating conservation has had a positive effect.

## Introduction

Historical deforestation of mangrove forests, coupled with their high carbon densities^1^, make it probable that land cover change (LCC) in mangroves has caused substantial carbon emissions over the past century^2–4^. The past 50 years has seen extensive deforestation of mangrove ecosystems^2–4^, and their replacement with aquaculture, agriculture, plantation, and urban land cover types^5^. Research and advocacy over the past 2 decades has highlighted the global importance of mangroves and their carbon stocks to policy makers, and spurred major conservation and restoration programmes^6,7^. Yet, despite considerable attention on mangrove carbon stocks and deforestation rates^3,8,9^, it remains unknown how changes in mangrove extent have impacted net changes in the global mangrove carbon stock over this period.

Previous research into mangrove carbon stock change has quantified the amount of carbon at risk of emission due to deforestation, by overlaying carbon stock maps with regions of forest loss^8,9^. However, it has not previously been possible to estimate net changes in mangrove carbon stock at a global scale due to a lack of information on two key factors: (1) gains in mangrove carbon stock due to reforestation of former mangrove areas, or afforestation in new areas^8,9^, and (2) the proportion of mangrove carbon that is lost from the system following conversion to another land cover^9^. As a dynamic ecosystem located on the coast, mangroves experience high turnover, with both natural forestation and deforestation occurring^10^. In addition, anthropogenic mangrove afforestation and reforestation has been attempted through restoration and rehabilitation programmes, which have become increasingly popular with governments and non-governmental actors over the past two decades (though with mixed success)^6^. Mangrove forestation may therefore account for substantial gains in mangrove carbon. In regenerating mangroves and newly established patches, the accumulation of biomass and soil carbon is not instantaneous, but progresses as the ecosystem develops, until reaching the stock of an established mangrove forest^11,12^. The accumulation of carbon in a developing mangrove ecosystem can take longer than 50 years^12^. Similarly, it is important to consider the proportion of mangrove carbon that is lost after deforestation, because the process of LCC does not completely release all of the mangrove carbon from the ecosystem immediately^13^. While some of the carbon stored in plant biomass or soil is lost relatively rapidly after natural or anthropogenic deforestation, some carbon is lost at a much slower rate, or may remain indefinitely^9^. As little as 25% of the carbon stored in tree biomass and soil may be lost following mangrove deforestation^13^.

We quantified net changes in the global mangrove carbon stock between 1996 and 2016, by combining recently-released datasets describing mangrove forest extent in 1996 and 2016^14–17^, the proportion of carbon stocks lost after conversion from mangroves to other land cover types^13^, and the rate of accumulation of carbon stocks during forestation^18^. We quantified the carbon at risk of loss due to LCC (*D*), the amount of carbon that may eventually be present in the area of forested mangrove (*F*), the proportion of carbon lost from the system given the time since deforestation (*r*_*t*_), and the proportion of carbon accumulated by foresting mangrove given the time since forestation (*a*_*t*_). We then used these parameters to estimate the net loss in carbon stock resulting from mangrove LCC (*Dr*_*t*_ − *Fa*_*t*_) between 1996 and 2016.

## Results

### Global net loss of mangrove carbon stocks

The total area of mangrove in 1996 was 142,865 km^2^, and the area in 2016 was 136,717 km^2^ ^17^. Between 1996 and 2016, an estimated 8050.4 km^2^ of mangrove forest was deforested through natural or anthropogenic causes. However, this loss of mangrove cover was partially offset by forestation of 2243.3 km^2^ ^17^. The net loss in mangrove extent was thus approximately 5807.2 km^2^; equivalent to 4.0% of the 1996 area^17^. The rates of forestation and deforestation did not consistently increase or decrease over the study period but were highest between 2007 and 2010 (Supplementary Fig. 1). The overall annualised net loss rate was 0.2% of the 1996 extent per year.

**Fig. 1:**
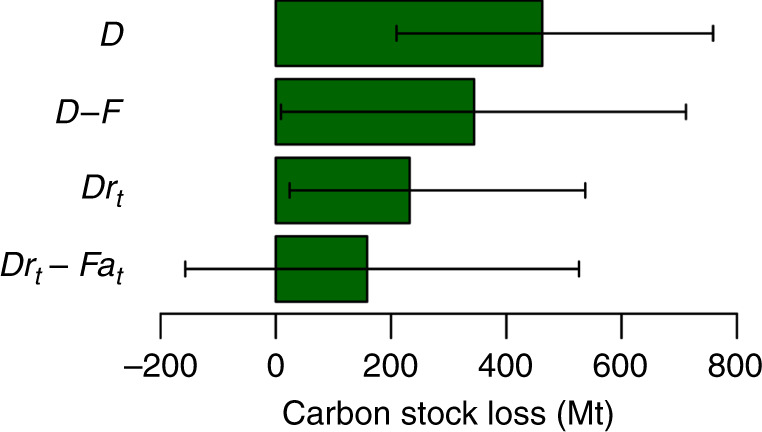
Global mangrove carbon stock loss between 1996 and 2016 under different estimation methods. Carbon at risk of loss due to mangrove deforestation (*D*), net loss of carbon estimated assuming 100% carbon loss and gain rates (*D* *−* *F*), loss of carbon due to deforestation after accounting for carbon loss rates (*Dr*_*t*_), net loss in mangrove carbon stock after accounting for forestation and carbon loss and accumulation rates (*Dr*_*t*_ − *Fa*_*t*_). Error bars indicate 95% bootstrap simulation confidence intervals (Methods).

The mangrove carbon stock in 1996 was 8627.6 Mt (95% CI: 4274.6–13,767.0 Mt). Between 1996 and 2016, the carbon stock at risk of loss due to mangrove deforestation (*D*) was 462.5 Mt (95% CI: 209.7–758.6 Mt; Fig. 1). The maximum carbon stock gain due to forestation (*F*) was 118.2 Mt (95% CI: 47.1–200.8 Mt). Without accounting for carbon loss and gain rates, the net loss of mangrove carbon stocks (*D* − *F*) was thus 344.3 Mt (95% CI: 9.0–711.6 Mt; Fig. 1). After accounting for carbon loss and gain rates over time, the carbon stock loss due to deforestation between 1996 and 2016 (*Dr*_*t*_) was 232.6 Mt (95% CI: 24.0–537.0 Mt), and gain due to forestation (*Fa*_*t*_) was 74.2 Mt (95% CI: 11.0–180.8 Mt). The net loss in global mangrove carbon stock (*Dr*_*t*_ − *Fa*_*t*_) was thus 158.4 Mt (95% CI: −156.8 to 525.9 Mt; Fig. 1). This estimated net loss of carbon stock is equivalent to 1.8% of the original carbon stock present in 1996. The lower confidence interval for *Dr*_*t*_ − *Fa*_*t*_ was negative, indicating a possible net gain in mangrove carbon stocks over the study period (Fig. 1).

### Spatial variation in mangrove carbon stock change

**Fig. 2:**
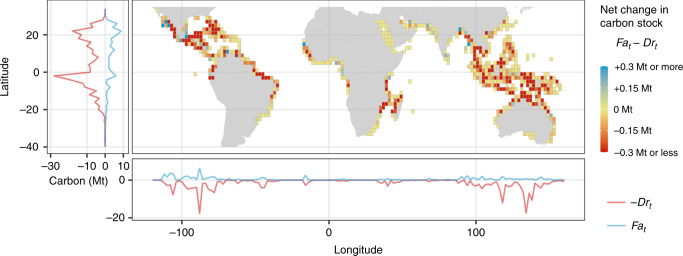
Spatial variation in net changes in mangrove carbon stock. Net change in mangrove carbon stock was quantified as (*Fa*_*t*_ − *Dr*_*t*_). Simulated values for each patch of mangrove gain and loss were aggregated to 2° grid resolution by taking the sum of all patches of mangrove gain and loss with centroids falling within each grid cell. Side panels indicate latitudinal and longitudinal variation in loss of mangrove carbon stocks due to deforestation (−*Dr*_*t*_) and gain due to forestation (*Fa*_*t*_). Pacific island and New Zealand mangroves not shown.

**Fig. 3:**
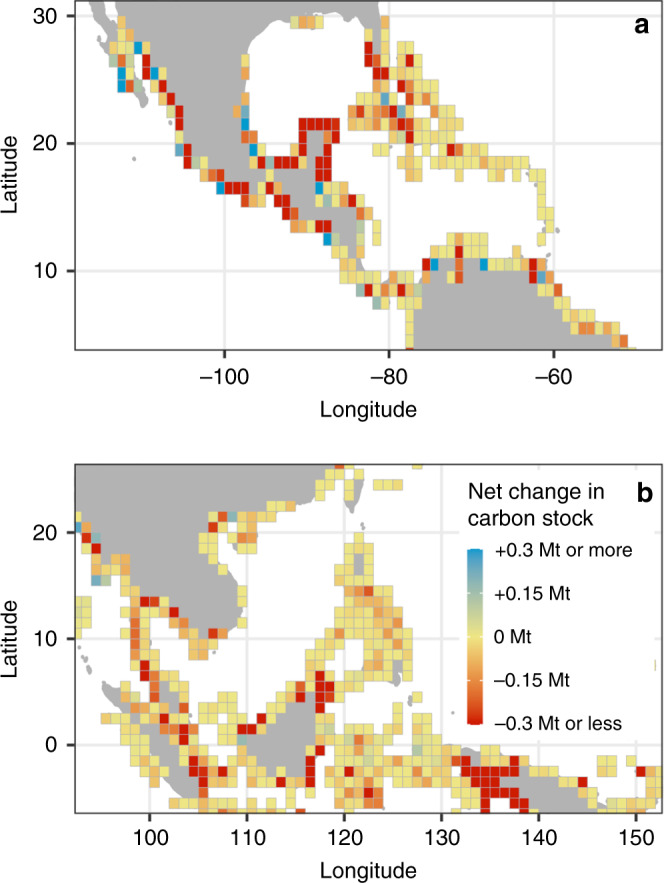
Net changes in mangrove carbon stock in two hotspots of change. Net change in mangrove carbon stock was quantified as (*Fa*_*t*_ − *Dr*_*t*_). **a** Central America and **b** Southeast Asia. Simulated values for each mangrove change patch were aggregated to a 1° grid resolution by taking the sum of all patches of mangrove gain and loss with centroids falling within each grid cell.

There was substantial spatial variation in both gains and losses of mangrove carbon stocks (Fig. 2). Substantial carbon losses occurred in the Caribbean and Southeast Asia, particularly on the islands of Borneo and Papua (Fig. 2). Gains of mangrove carbon were smaller, and only substantially outweighed losses in a few scattered regions across Africa, South Asia, and Central America (Fig. 2). A more detailed look at Central America and Southeast Asia indicates high spatial variability even within these regions, with large areas such as Panama, the Philippines, and southern China showing little change (Fig. 3a, b). There were high net losses of mangrove carbon localised to parts of Mexico, Borneo, and Papua (Fig. 3a, b), and net gains in part of Mexico and Myanmar (Fig. 3a).

## Discussion

Substantial mangrove forestation occurred between 1996 and 2016. Accounting for the resulting accumulation of carbon greatly reduced the estimated net loss in carbon stock (Fig. 1). The contribution of mangrove forestation in reducing net carbon stock loss is important in light of an international goal to increase global mangrove area by 20% by 2030^6^. Given the currently available data, it is not possible to distinguish between the relative contributions of natural mangrove regeneration and forest expansion, compared to that of anthropogenic restoration and rehabilitation. Climate change has allowed mangroves to invade temperate saltmarsh ecosystems, thus leading to a substantial poleward range expansion^19^. This expansion may partially explain net gains in mangrove carbon stocks in Mexico (Fig. 3a). Similarly, natural regeneration of previously deforested mangroves has occurred in regions where economic change has led to land abandonment^20^, and natural colonisation of newly suitable terrain has occurred in areas experiencing high aggradation^10^. In terms of anthropogenic reforestation and afforestation, a substantial investment has been made in recent years, although often without long-term success^6,21^. Despite high rates of failure due to poor restoration design or choice of location, some projects have successfully used ecological principles and conducted appropriate post-planting care and monitoring, to allow mangrove establishment^21^. For example, substantial efforts to conserve and replant mangroves may have led to the low net loss in carbon stock in southern China^22^ (Fig. 3b).

Accounting for the proportion of carbon lost following LCC also substantially reduced the estimate of global net carbon stock loss (Fig. 1). Changes in carbon stock during processes of mangrove deforestation and conversion are complex to monitor and spatially variable depending on the local soil and plant characteristics^23^. A recent synthesis of the literature has provided a valuable resource in accounting for losses of carbon^13^, and may encourage future studies to better quantify *r*_*t*_. The most recent previous analysis of changes in global mangrove carbon stock reported maximum potential emissions due to mangrove deforestation (equivalent to *D* in our analysis), alongside an estimate made assuming an arbitrary *r*_*t*_ value of 25% carbon loss^9^. Our improved estimate of net loss in global mangrove carbon stock due to LCC (*Dr*_*t*_ *−* *Fa*_*t*_) was 34.2% of *D* (Fig. 1), highlighting the critical importance of quantifying mangrove forestation and accounting for proportional carbon loss and accumulation after LCC. *D* provides an overestimate of actual carbon stock losses^9^, making the values unreliable for use in global or national carbon accounting initiatives.

If we were to assume that all of the net loss in mangrove carbon stock (*Dr*_*t*_ *−* *Fa*_*t*_) was released into the atmosphere as carbon dioxide, the total emission between 1996 and 2016 would equate to 580.4 Mt (95% CI: −574.6 to 1927.0 Mt). This is not an insignificant emission, however, it is important to put this value into context given the continued high levels of interest in mangrove research, conservation, and restoration^2,6,24^. Over the same 20 year period as used in this study, 580.4 Mt of carbon dioxide is equivalent to 0.6% of the emissions from LCC, and less than 0.1% of the total global CO_2_ emissions^25^. This small contribution highlights that while mangroves can hold high densities of carbon^1^, their small footprint means that researchers must be careful not to oversell the significance of mangrove blue carbon for climate change management and mitigation^7^.

The small net change in mangrove carbon stock is also a result of a relatively low mangrove deforestation rate (mean 0.3% per year between 1996 and 2016), coupled with substantial mangrove forestation, resulting in a mean net loss rate of 0.2% per year. This net mangrove deforestation rate is relatively low in comparison with some other tropical ecosystems; for example, between 1990 and 2010, peatland forests experienced a net loss of 54,000 km^2^ in Southeast Asia alone, equivalent to a net loss rate of 3.7% per year^26^. High rates of net loss have also been reported in blue carbon ecosystems, such as seagrasses^27^. The low net rate of mangrove loss suggests that mangrove awareness-raising and conservation efforts have found some success, at least in the period between 1996 and 2016. There has not been a consistently declining rate of deforestation or consistently increasing rate of forestation between 1996 and 2016 (Supplementary Fig. 1), although recent research using a different remote sensing method has shown a declining rate of total mangrove area loss between 2000 and 2016, and declining contribution of direct anthropogenic drivers of deforestation over the same period^28^. Longer-term records of mangrove cover and deforestation rate are not accurate enough to estimate the point in time when mangrove deforestation rates slowed globally^29^. If mangroves are indeed a conservation success story^30^, then future research must identify when this turning-point happened, and how it was achieved. Given the apparent success of mangrove conservation efforts^30^, key lessons learnt from this experience could be applied to other ecosystems.

Mangrove conservation and restoration still requires considerable effort and investment, to maintain the low rates of net loss in mangrove carbon stocks, and perhaps increase those stocks through restoration^24^. The high densities of carbon present in mangroves provide good carbon returns per unit area conserved, and the low agricultural value of the land for many crops may make mangrove forests a suitable candidate ecosystem for carbon investment schemes^31^, although this is context-dependent and uncertain^32^. Furthermore, mangroves provide a range of other ecosystem services, such as protecting coastlines from natural disasters^33^, supporting fisheries^34^, and supporting recreation and tourism^35^. These additional ecosystem services may provide further justification for conservation and restoration efforts.

Considerable uncertainty surrounds the estimation of net losses in global carbon stock (Fig. 1). Such uncertainty is almost inevitable when synthesising multiple global datasets, but the confidence intervals reported here are not large enough to cast doubt on the main conclusion of the study. Converting the upper 95% confidence interval of carbon stock loss to equivalent potential carbon dioxide emissions would give an estimate of 1927.0 Mt CO_2_—equivalent to 0.3% of total global CO_2_ emissions^25^. Therefore, even our least conservative estimate represents a relatively low rate of net loss in mangrove carbon stocks between 1996 and 2016. Furthermore, the lower 95% confidence interval for net carbon stock loss was negative, indicating that mangrove carbon stocks may in fact have increased over the study period (Fig. 1).

There are additional sources of uncertainty involved in quantifying net changes in mangrove carbon stock, that are not incorporated in our simulation or in the generation of the confidence intervals reported here (Fig. 1). First, we focused on soil organic carbon and biomass carbon stocks, due to the global availability of these data^36,37^. Other stocks of mangrove carbon are held in inorganic form in sediments, and in dead biomass; these two stocks may account for around 25% of the total held in mangrove forests^38^. Second, we focused on estimating changes in mangrove carbon stocks based on changes in mangrove cover, because deforestation has been highlighted as a potential major source of global carbon emissions^9,39^. Smaller-scale degradation of mangroves through timber collection or pollution is also likely to degrade carbon stocks, but has not been quantified at a global scale due to challenges in observing these processes using remote sensing^24^. Third, our estimates are built on the existing body of mangrove carbon research, and thus may be sensitive to disciplinary biases and errors in this literature. Global mapping of mangrove carbon stock densities are likely biased to high-quality mangrove sites, while large areas of mangrove forest may be disturbed or degraded through natural or anthropogenic mechanisms^37^. If on-the-ground carbon stock densities are commonly lower or higher than these potential estimates, we might expect deforestation events to result in smaller or greater losses of mangrove carbon stock. Similarly, if reforesting mangroves develop into disturbed or degraded rather than high-quality forests, gains in mangrove carbon stock may differ from our estimates. Recent research has suggested that stocks of soil organic carbon may have been significantly over-estimated in past research, due to the use of unsuitable conversion factors when translating laboratory measurements to carbon content values^38^. We conducted a sensitivity analysis to quantify the potential impact of this error on net changes in mangrove carbon stock in Southeast Asia, finding that correcting for this miscalculation may result in 35% lower estimates of net mangrove carbon stock loss in the region (Supplementary Methods 1).

This study focused on quantifying net changes in mangrove carbon stock, but it is more complex to estimate the total net carbon emissions following mangrove-related LCC. First, in cases of mangrove forestation, it would be necessary to account for pre-mangrove carbon stocks which may be lost or added to following mangrove forestation. This may be particularly important in the case of mangrove encroachment into mud flat or seagrass ecosystems, which can store substantial (although typically lower than mangrove) carbon stocks^40,41^. Second, for all forms of mangrove LCC, there are changes not only in the stock of carbon, but also in the rates of carbon flux^13^. Future work may build upon the simulation framework developed here, to incorporate developments in our scientific understanding of mangrove carbon stocks and fluxes, and improve global estimation of net changes in mangrove-related carbon emissions.

Here, we provide an estimate of recent net changes in the global mangrove carbon stock due to LCC, and suggest future avenues of research to further develop this approach. Net losses of global mangrove carbon stocks highlight the high densities of carbon stored in this ecosystem, but are low in the context of global emissions from LCC and other sectors. The low mangrove deforestation rate and substantial forestation imply that global efforts to conserve and restore mangroves have had some success.

## Methods

### Overall simulation approach

We estimated net changes in soil organic, above-ground and below-ground living biomass carbon stocks due to mangrove-related LCC that occurred globally between 1996 and 2016, for the year 2016. We did not estimate carbon sequestration because while sequestration rates in mangrove forests are higher than in many other ecosystems, there is only limited capacity for this process to substantially impact global carbon fluxes due to the small area of mangrove forest^7,32,39^. In addition, global high-resolution maps of mangrove carbon sequestration rates are not available.

Uncertainty in estimates of the area of mangrove loss and gain, carbon stock density, date of deforestation or forestation, proportional carbon stock degradation due to deforestation, and proportional carbon stock accumulation due to forestation, was carried forward using a bootstrap simulation method, through which 1000 replications were used to generate median estimates and 95% confidence intervals. A summary of the sources of data, simulation parameters, and modelling of parameter variability is provided in Supplementary Table 1. For each bootstrap iteration, the area of mangrove gain and loss, carbon stock density, *r*_*t*_, and *a*_*t*_ values were simulated within each patch of mangrove gain or loss. The *D*, *F, Dr*_*t*_, and *Fa*_*t*_ values for each patch were then calculated. The median value of the 1000 replicates was used as the estimate. The 2.5 and 97.5 percentiles of the 1000 simulation estimates of change in carbon stock were calculated, thus corresponding to bootstrap 95% confidence intervals for each of the estimated losses or gains in carbon stock within each patch. A comparable bootstrap method was applied to simulate carbon stocks in 1996 for each patch of mangrove present at that time.

In addition to the sources of uncertainty incorporated in the bootstrap simulation, we made several key methodological decisions that could be expected to impact the conclusions of the study. We conducted four sensitivity analyses to quantify the impacts of such decisions. The sensitivity analyses were conducted only for the region of Southeast Asia, because one sensitivity analysis required detailed and spatially explicit data on the replacement land uses following mangrove deforestation, which are only available with the necessary categorisation for Southeast Asia^5^. Methodological details and results of the sensitivity analyses are included in Supplementary Methods 1.

### Mangrove areal extent

We used the Global Mangrove Watch (GMW) datasets of mangrove cover to quantify deforestation and forestation^14–17^. While other global mangrove map products exist for specific years^42,43^, GMW provides maps of extent from multiple years, allowing temporal comparison. The mangrove extent in 1996 and 2016 was mapped using the GMW data products, which are derived from ﻿ALOS PALSAR and Landsat satellite-borne sensor data^14–16^. Mangrove-related LCC was defined either as a conversion from mangrove to another form of land or water cover between the 1996 and 2016 datasets (deforestation), or vice versa (forestation). Any change in mangrove cover that was reversed before the end of the study period was therefore not captured. Areas of overlapping and nonoverlapping mangrove extent were compared between these dates to quantify mangrove present in 1996 that was not present in 2016, mangrove present in 2016 that was not previously present in 1996, and areas of no change in mangrove cover between 1996 and 2016. For each patch of mangrove cover in 1996, gain, and loss, the area was calculated under the Eckert VI equal-area projection. This projection was used to calculate the area of mangrove and mangrove change polygons only, and all other analyses were conducted using the World Geodetic System (WGS) 1984 projection^17^.

The GMW mapping of mangrove forest has an error rate^15^, leading to quantifiable uncertainty over the presence of mangroves at each location in 1996 and 2016. Accuracy statistics have not been published for each year in the GMW dataset^17^, so we assumed that all years had an identical accuracy to the best-documented year, which is 2010^15^. Published error rates correspond to individual pixels in the original GMW dataset, so we modelled uncertainty at this spatial scale. For each pixel of recorded mangrove gain or loss, there is a probability that it is an erroneous, false positive example of gain or loss. For each pixel of mangrove or non-mangrove that is recorded in GMW as being the same in 2016 as in 1996, there is a similar probability of error—a false-negative case of gain or loss. While false positive gain and loss errors can be quantified fully based on the available information, we were not able to incorporate false negative errors in the simulation (Supplementary Methods 2). However, false-negative errors can be expected to impact estimates of forestation and deforestation area almost equally, while false-positive errors are biased toward a greater effect on estimates of deforestation (Supplementary Methods 2). For these reasons we incorporated only false positive classification uncertainty into the bootstrap simulation and generation of carbon stock change confidence intervals. Uncertainty in the areal extent of mangroves was not incorporated into the bootstrap simulation for the estimate of carbon stocks in 1996. As the uncertainty estimation for areal extent change does not include false negative classification errors, we do not report confidence intervals for area change statistics, and report only the median estimates from the bootstrap replicates.

For areas of mangrove loss and gain, we incorporated the probability of false positive recording of loss and gain into the simulation. For each pixel within each patch of mangrove gain, we simulated whether it was actually not mangrove in 1996 according to the misclassification error rate for non-mangrove (Supplementary Table 2), and whether it was truly mangrove in 2016 according to the misclassification error rate for mangrove (Supplementary Table 2). The simulated number of gain pixels in each patch was thus calculated as the number of pixels that were simulated to have been both not mangrove in 1996, and mangrove in 2016. Similarly, for each pixel recorded as mangrove deforestation, there is a probability that it was a false positive example of loss. For each pixel within each patch of mangrove loss, we simulated whether it was truly mangrove in 1996 according to the misclassification error rate for mangrove (Supplementary Table 2), and whether mangrove was truly absent in 2016 according to the misclassification error rate for non-mangrove (Supplementary Table 2). The simulated number of loss pixels in each mangrove patch was thus calculated as the number of pixels that were simulated to have been mangrove in 1996, and not mangrove in 2016.

### Carbon stock density

Spatial patterns in mangrove carbon densities were quantified using previously published datasets of soil carbon to 1 m depth^36^, and above- and belowground tree biomass carbon^37^. Both datasets are derived from systematic reviews of the literature, so may be biased towards relatively high-quality mangrove forests, rather than those that have experienced some natural or anthropogenic disturbance^37^. The resulting maps of carbon stock density are therefore likely to represent an upper estimate for the potential carbon stock density at each location^37^. As the dates and resolutions of these mangrove carbon datasets differed from the GMW mangrove extent, per hectare carbon densities were extracted for each patch of mangrove extent in 1996, gain, and loss of mangrove. Where possible, we extracted mean carbon densities for the 0.05 degree grid cell (approximately 5 km) in which the centre of the mangrove patch coincided (Supplementary Table 3). Where data did not coincide at this resolution, 0.5° grid cells (approximately 50 km) were used (Supplementary Table 3). Any remaining data gaps were filled using the global mean carbon stock density (Supplementary Table 3).

Uncertainty in the estimate of carbon stock was modelled as a normally distributed random variable, with the mean value taken as the reported carbon stock density extracted from the published map layers^36,37^, and the standard deviation of the distribution taken as the reported root mean squared error (RMSE) between the model predictions and validation data^36,37^. The RMSE for soil carbon is reported in the study as 109 Mg per hectare^36^. The RMSE for aboveground biomass carbon was calculated as 104.1 Mg per hectare, based on a plot of observed versus predicted values digitised from the original study^37^ (Supplementary Fig. 2). The soil and biomass carbon stock densities for each patch of mangrove in 1996, patch of mangrove loss, and patch of mangrove gain were simulated, and values of less than zero were replaced with zero, to avoid negative carbon densities.

### Loss and accumulation of mangrove carbon

After mangrove deforestation, carbon is lost gradually over a period of time, with biomass carbon typically depleting more rapidly than carbon stored in soils^13^. We modelled temporal losses of soil carbon stocks according to a previously-published meta-analysis of the proportion of the reference carbon stock (*r*_*t*_) lost over time^13^. For losses of biomass carbon stocks, we used a meta-analysis of temporal changes in the proportion of the reference tree diameter as a proxy for biomass carbon stock, because a meta-analysis of temporal changes in biomass carbon stock was not available^13^. The shape of these temporal *r*_*t*_ relationships can be observed in Supplementary Fig. 3a, b. The approximate date of mangrove deforestation was quantified by cross-referencing several dates from the GMW dataset to establish the dates of presence and absence^17^. We cross-referenced the dates of 1996, 2007, 2010, and 2016 to identify the dates of mangrove presence and absence at each location.

Uncertainty in proportional losses of carbon due to mangrove deforestation was incorporated in two ways. First, there is uncertainty in the date of mangrove deforestation since the most recent observed date of presence. To model uncertainty in the date of deforestation we used a uniform distribution to select a date between the most recent date of observed mangrove presence and the oldest date of observed mangrove absence. Second, there is uncertainty in the relationship between the time since deforestation and proportion of mangrove carbon lost, quantified as the error present in the regression models (Supplementary Fig. 3a, b). We simulated the projected proportions of mangrove carbon remaining as a function of the length of time since deforestation (2016—date of deforestation), accounting for the error inherent in each linear model (Supplementary Methods 3).

As mangrove forests grow, they typically accumulate carbon in soil and tree biomass stocks, until reaching the value held by the reference community^12,44^. This process can be slow, taking from 20 to more than 50 years^12,44^. At a given point in time before the climax community is reached, the mangrove ecosystem contains a proportion of the value held in the climax community (*a*_*t*_). The whole-ecosystem carbon accumulation curve for afforesting mangroves was estimated using data taken from a meta-analysis of blue carbon ecosystem restoration^18^, that we used to estimate the proportion of the reference ecosystem carbon accumulated following restoration (Supplementary Methods 4). The shape of the temporal relationship can be observed in Supplementary Fig. 3c. We used this general relationship describing restoration of all blue carbon ecosystems, because a mangrove forestation-specific meta-analysis is not currently available. To assess the impacts of this selection on the study findings, we also conducted a sensitivity analysis using data from two case studies of mangrove soil carbon and biomass accumulation in foresting mangroves (Supplementary Methods 2). The approximate date of mangrove forestation was quantified by cross-referencing several dates from the GMW dataset to establish the dates of presence and absence^14–16^. We cross-referenced the dates of 1996, 2007, 2010, and 2016 to identify the dates of mangrove presence and absence at each location.

Uncertainty in gains of carbon due to mangrove forestation was incorporated in two ways. First, there is uncertainty in the date of mangrove forestation since the most recent observed date of presence. To model uncertainty in the date of forestation we used a uniform distribution to select a date between the most recent date of observed mangrove absence and the oldest date of observed mangrove presence. Second, there is uncertainty in the relationship between the time since deforestation and proportion of mangrove carbon lost, quantified as the error present in the meta-analytic regression model (Supplementary Fig. 3c). We simulated the projected proportions of mangrove carbon remaining as a function of the length of time since deforestation (2016—date of deforestation), accounting for the error inherent in each linear model^45^ (Supplementary Methods 3).

We estimated *D*, *F*, *r*_*t*_, and *a*_*t*_ for each patch of mangrove gain and loss between 1996 and 2016. These data were then used to quantify four indicators of net change in mangrove carbon stocks, to evaluate the sensitivity of estimation to the inclusion or exclusion of afforestation and remnant carbon processes. The first indicator estimated the maximum carbon stock at risk of loss due to deforestation (*D*), following the approach used in the most recent global estimate of potential mangrove carbon emissions^9^. The second indicator estimated net loss of carbon assuming 100% carbon loss and gain rates (*D* − *F*). For the third indicator, we estimated the carbon stock loss due to deforestation but accounting for remnant carbon (*Dr*_*t*_)^8^. Finally, the fourth indicator estimated net changes in mangrove carbon stock between 1996 and 2016 accounting for both forestation and proportional accumulation and loss rates of carbon following LCC (*Dr*_*t*_ − *Fa*_*t*_). For mapping of spatial variability in net gains and losses of mangrove carbon stocks, we quantified the net change in mangrove carbon stock (*Fa*_*t*_ − *Dr*_*t*_) by summarising all patches of mangrove gain and loss with their centroids located in cells across a global grid (Figs. 2 and 3).

## Supplementary information

Supplementary Information

## Data Availability

All original datasets used in this study are available from the respective references, with most being open access. The generated data that form the results of this study are available from the corresponding author upon reasonable request.

## Code availability

Code for the simulation of new values from a regression model is available in Supplementary Information 3. No other new algorithms were developed in this study. Code used to conduct the analysis is available from the corresponding author upon reasonable request.
