# Supplementary material for: Quantifying net loss of global mangrove carbon stocks from 20 years of land cover change
Source: Nat Commun. 2020 Aug 26;11:4260. doi: 10.1038/s41467-020-18118-z (PMC7450071; doi:10.1038/s41467-020-18118-z)
Supplement: Supplementary file 1 — Supplementary Information [file 41467_2020_18118_MOESM1_ESM.pdf]

Supplementary Information for “Quantifying net loss of global mangrove carbon stocks from 20 years of land cover change” by Richards et al.

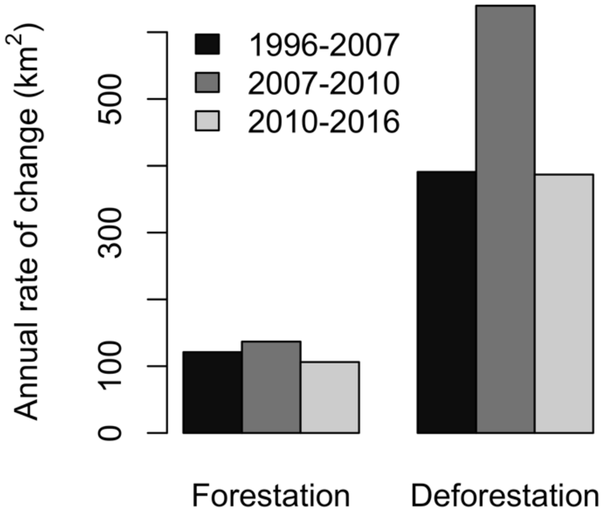

Supplementary Fig. 1. Annualised rates of gains and losses in mangrove area between 1996 and 2016, for three time periods. Rates of change correspond to net forestation and deforestation recorded between the dates of 1996 and 2016, rather than time interval-specific forestation and deforestation events.

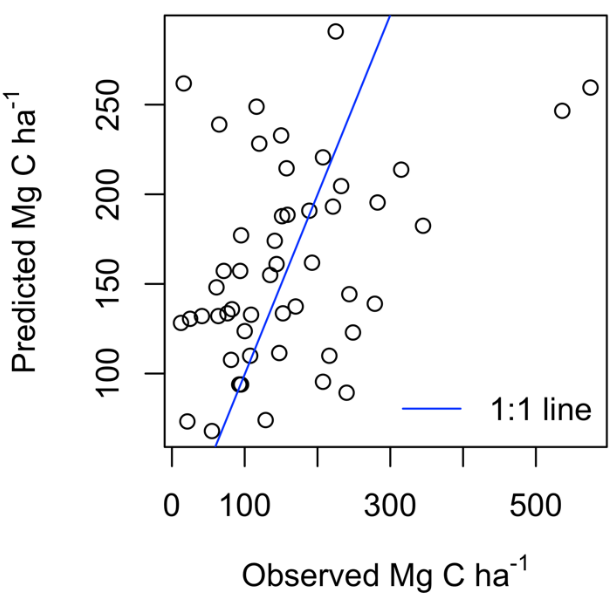

Supplementary Fig. 2. Comparison of observed against predicted values of aboveground biomass taken from the validation performed in the global mangrove biomass mapping study[1].

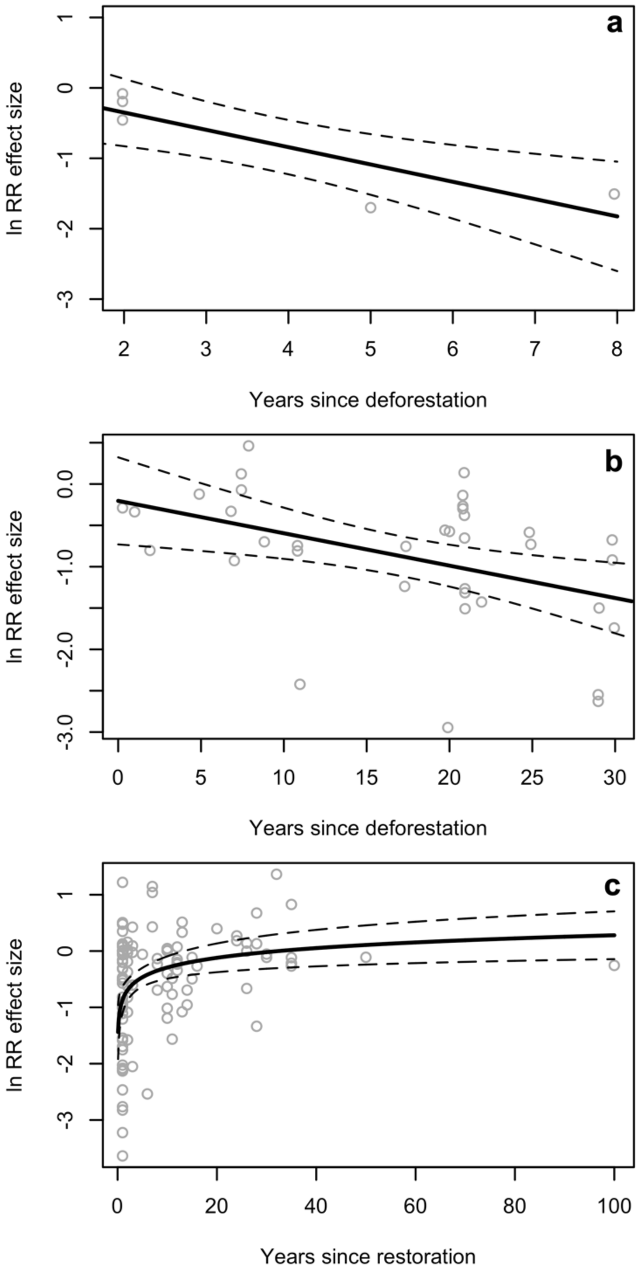

Supplementary Fig. 3. Temporal patterns in losses and gains of mangrove carbon stocks. (a) Losses of tree diameter as a proxy for losses in biomass carbon, following mangrove deforestation events. (b) Losses of soil carbon stocks following mangrove deforestation events. (c) Gains of carbon following restoration of blue carbon ecosystems. All y axes are transformed to the natural log response ratio of proportional difference between the natural reference ecosystem and the deforestation (a:b) or restoration (c) samples. Black solid lines indicate regression lines, with dashed lines indicating one standard error.

Supplementary Table 1. Outline of datasets used in bootstrap simulation of changes in global mangrove carbon stock, and summary of additional sensitivity analyses conducted.

Parameter	Source	Simulation	Sensitivity analyses
Area of mangrove forestation and deforestation	GMW mapping of mangrove extent in 1996 and 2016[2–4].	Forestation and deforestation was modelled for each pixel within each mangrove patch as a binomial variable, taking into account the probability of false positive errors[2].	None.
Soil carbon density	Published global mangrove soil carbon map[5].	The mangrove soil carbon density within each polygon was drawn from a normal distribution with mean taken to be the predicted value and standard deviation to be the reported RMSE of prediction[5].	Recent research has suggested that mangrove soil carbon densities may have been overestimated in the past[6]. We ran a simulation using corrected estimates according to this study (Sensitivity Analysis 1, see Supplementary Methods 1).
Biomass carbon density	Published global mangrove biomass map[1].	The biomass density within each polygon was drawn from a normal distribution with mean taken to be the predicted value and standard deviation to be the reported RMSE of prediction[1].	None.
Date of mangrove loss	GMW mapping of mangrove extent in 1996, 2007, 2010 and 2016[2–4,7].	The date of mangrove loss was simulated as a uniformly-distributed date between the most recent date of observed mangrove presence and the oldest date of observed mangrove absence.	None.
Date of mangrove gain	GMW mapping of mangrove extent in 1996, 2007, 2010 and 2016[2–4,7].	The date of mangrove gain was simulated as a uniformly-distributed date between the most recent date of observed mangrove absence and the oldest date of	None.

		observed mangrove presence.	
Proportion of carbon stock lost (r_t)	Meta-analysis of temporal patterns in degradation of aboveground biomass and soil carbon stock[8].	The proportion of carbon stock lost was simulated from the distribution corresponding to the fitted meta-analysis model[8].	Conversion of mangroves to different land cover types can have different values for r_t. Temporal patterns of r_t have not been quantified across different land cover change types, but static values have been quantified[8]. We conducted two sensitivity analyses, one using overall static mean r_t values (Sensitivity Analysis 2, see Supplementary Methods 1), and one using specific static r_t values for different types of mangrove conversion (Sensitivity Analysis 3, see Supplementary Methods 1).
Proportion of reference carbon stock accumulated (a_t)	Meta-analysis of temporal patterns in accumulation in whole-ecosystem blue carbon stock following restoration, used as an indicator for mangrove forestation[9].	The proportion of carbon stock accumulated was simulated from the distribution corresponding to a fitted meta-analysis model (Supplementary Methods 4)[9].	The meta-analysis relationship used here was general for all blue carbon ecosystems, not specific to mangroves. We conducted a sensitivity analysis using mangrove-specific a_t values from two case studies[10,11] (Sensitivity Analysis 4, see Supplementary Methods 1)

Supplementary Table 2. Error matrix of mangrove classification from GMW 2010[2].

	Probability of actually being mangrove	Probability of actually being non-mangrove
Classified as mangrove	0.975	0.025
Classified as non-mangrove	0.033	0.967

Supplementary Table 3. Methods of assignment of carbon stock densities for mangrove patches.

	Mangrove patches with data at 5 km resolution	Mangrove patches with data at 50 km resolution	Mangrove patches with data taken from global mean
Mangrove area in 1996 aboveground carbon	696,109	4,252	543
Mangrove area in 1996 soil carbon	642,383	50,160	8,361
Mangrove deforestation and forestation aboveground carbon	1,735,611	8,257	802
Mangrove deforestation and forestation soil carbon	1,670,741	65,211	8,718

Supplementary Methods 1. Sensitivity analyses

We conducted four sensitivity analyses to quantify the impacts of separate methodological decisions on the conclusions of the study. The sensitivity analysis was conducted across 100 bootstrap replicates, for all patches of mangrove gain and loss within the region of Southeast Asia (defined as the Association of Southeast Asian Nations member states, plus Timor-Leste). Only the region of Southeast Asia was analysed because one of the sensitivity analyses required data on the replacement land covers following mangrove deforestation, which are only available with categories comparable to the meta-analysis of r_t values in this region[8,12]. While there is now a global mapping of the broad-scale land cover types driving mangrove deforestation[13], this categorisation does not map onto the commodity-level categories used in the meta-analysis[8]. In addition to the four sensitivity analysis simulations, we conducted a baseline simulation using identical methods to those followed in the main study, to act as a reference point.

Sensitivity Analysis 1

We investigated the sensitivity of the conclusions to systematic errors in the way that mangrove soil organic carbon stocks have been quantified in past research[6]. A recent study by Ouyang and Lee found that the conversion factor used to translate soil loss-on-ignition (*LOI*) data to organic carbon content (*OC*) may have been incorrectly defined in past research[6]. The soil organic carbon dataset we used in this study applied a constant LOI/ OC conversion factor of $OC = 0.5 \times LOI$ [5], while the Ouyang and Lee study found that relationship may actually better described as a polynomial line[6] (Supplementary Fig. 4). It was not feasible to re-analyse the existing literature and repeat a synthesis and mapping exercise of mangrove soil carbon stocks globally. We therefore developed a method to approximate *OC*, in order to estimate an Ouyang and Lee-corrected soil organic carbon stock density for each patch of mangrove. The following approximation makes a number of significant assumptions, so we proceeded with the previously-published global mangrove soil organic carbon dataset for the purpose of the main study. However, this approximation is conducted as a sensitivity analysis to explore the potential impacts that applying the Ouyang and Lee correction might have on estimation of global mangrove carbon stock loss. As future empirical research incorporates the Ouyang and Lee conversion factor, more data will become available to repeat global mapping and modelling efforts.

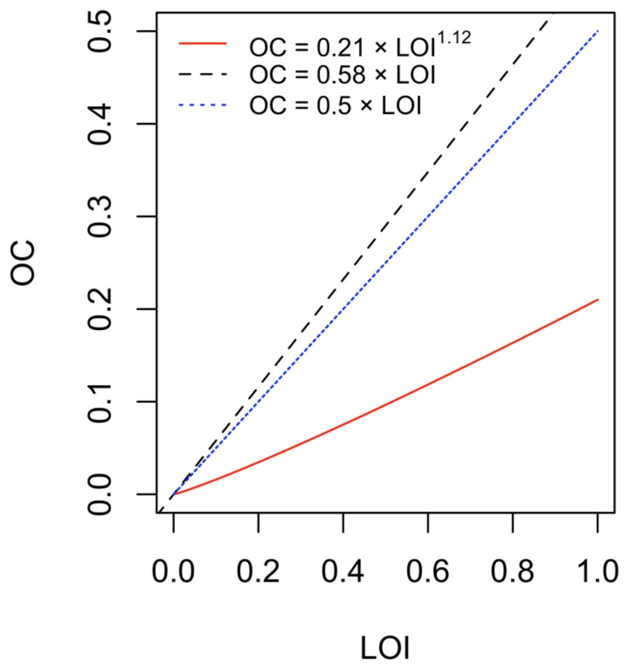

Supplementary Fig. 4. Conversion factors between *LOI* and *OC*, including those commonly used in previous literature, the relationship used in the global soil mapping analysis we used to provide soil carbon stock data[5],and the relationship proposed by Ouyang and Lee[6].

To apply the Ouyang and Lee correction ideally requires data on the organic carbon content of the soil (*OC*); information that is not mapped spatially in the global mangrove soil carbon dataset we used here[5]. We therefore developed a method to approximate *OC*, in order to estimate an Ouyang and Lee-corrected soil organic carbon stock. The carbon stock density (*CD*) is typically estimated as;

$$CD = OC \times BD \times (1-CF)$$

With *BD* being the bulk density of the soil, and *CF* being the fraction of coarse matter present. We simplify this equation by assuming that *CF* is equal to zero, and that the bulk density *BD* can be estimated from the organic carbon content *OC*, following the empirically-estimated equation[5];

$$BD = 0.0906 + 0.8757 \times \exp(-0.0786 \times OC) + 0.6258 \times \exp(-1.0975 \times OC)$$

Combining these equations allows us to solve for *OC* based a known value of *CD*;

$$CD = OC \times (0.0906 + 0.8757 \times \exp(-0.0786 \times OC) + 0.6258 \times \exp(-1.0975 \times OC))$$

This equation was solved numerically for *OC* using the uniroot function in the R statistical programming language[14], for the range of *CD* values present in the soil carbon stock dataset[5]. The resulting relationship between *CD* and *OC* was modelled as a linear regression (Supplementary Fig. 5; Supplementary Table 4).

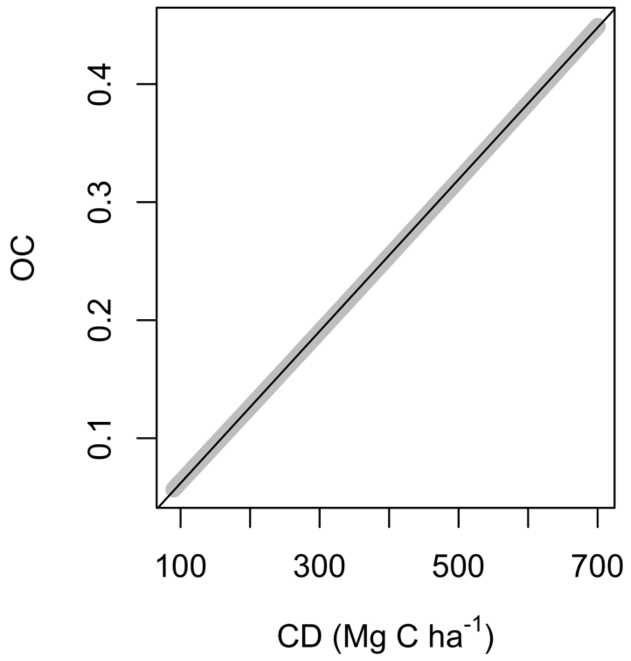

Supplementary Fig. 5. Modelled relationship between soil organic carbon density *CD* and soil organic carbon content *OC*. Grey points indicate *OC* values estimated for sequential *CD* values between 0.009 and 0.07.

Supplementary Table 4. Linear regression relationship between *CD* and *OC*. R^2 for the regression model is 1.

Coefficient	Estimate	Standard error	t	p
Intercept	-0.0024	< 0.001	-44.8	< 0.001
CD	0.0006	< 0.001	5197.9	< 0.001

Once the organic carbon content for the global mangrove soil carbon dataset (OC_s) was known for each mangrove polygon, we converted it to the Ouyang and Lee-corrected value (OC_o). This correction was made by cross-referencing the difference between the $OC = 0.5 \times LOI$ line, which was the conversion factor used in the global soil carbon mapping study[5], and the Ouyang and Lee line at $OC = 0.21\times LOI^{1.12}$ (Supplementary Fig. 4). We modelled the difference between the original and Ouyang and Lee values of *OC* as a generalised linear model using a quasibinomial error structure (Supplementary Fig. 6; Supplementary Table 5). We used this modelled relationship to estimate the soil organic carbon content of each mangrove patch under the Ouyang and Lee correction OC_o. We then converted these values back to estimate the soil organic carbon stock density after accounting for the Ouyang and Lee correction (CD_o), by reversing the linear regression equation for the line shown on Supplementary Fig. 5. These Ouyang and Lee-corrected estimates of soil organic carbon stock density were then used to re-run the modelling of net changes in mangrove carbon stock. All other parameters were maintained at the values used in the baseline study.

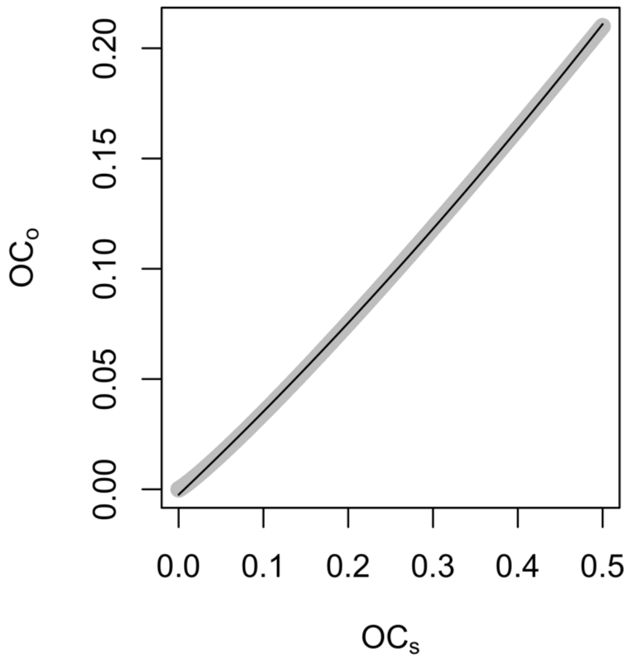

Supplementary Fig. 6. Relationship between the soil organic carbon content estimated using the Ouyang and Lee correction (OC_o) and the soil organic carbon content estimated using the $OC = 0.5 \times LOI$ line (OC_s). Grey points indicate OC_o values estimated for sequential OC_s values between 0 and 0.5

Supplementary Table 5. Linear regression relationship between OC_s and OC_o. Multiple R^2 for the regression model is 1.

Coefficient	Estimate	Standard error	t	p
Intercept	-0.002	< 0.001	-160.6	< 0.001
OC_s	0.365	< 0.001	2596.7	< 0.001
OC_s^2	0.125	< 0.001	457.2	< 0.001

Sensitivity Analysis 2 and Sensitivity Analysis 3

We investigated the sensitivity of the study conclusions to different methods of estimating the proportion of mangrove carbon lost following mangrove deforestation (r_t). In the main study, we used temporally-varying r_t values taken from a systematic meta-analysis[8]. These temporally-varying estimates represent the overall r_t values calculated across all types of mangrove conversion. However, the same meta-analysis showed that conversion of mangroves to different replacement land covers can have different impacts on the loss of carbon stocks[8]. Estimates of r_t for common types of post-mangrove land cover types, and confidence intervals, are provided in the meta-analysis[8], but we did not use these values in the study, firstly because they were only available as static mean values, rather than temporally-varying values[8]. Secondly, data on replacement mangrove land cover is not available globally at the required typological resolution[12].

In Sensitivity Analysis 2 we used static overall mean r_t values extracted from the meta-analysis, and in Sensitivity Analysis 3 we used static land cover change-specific r_t values. The r_t values were randomly generated for each polygon using a uniform distribution within the 95% confidence intervals reported in the meta-analysis[8]. Data on the replacement land covers that followed mangrove deforestation

between 2000 and 2012 were extracted from a previously-published study of Southeast Asia[12]. We assumed that all deforestation within each 2 degree grid cell was caused by the most commonly-occurring form of replacement land cover within the grid cell[12]. Grid cells that did not coincide with any recorded replacement land covers were assigned overall mean r_t values. The alternative r_t values were then used to re-run the modelling of net changes in mangrove carbon stock. All other parameters were maintained at the values used in the baseline study.

Supplementary Analysis 4
We investigated the sensitivity of the study conclusions to an alternative method of estimating the proportion of mangrove carbon accumulated in the ecosystem following mangrove forestation (a_t). In the study, we used a whole-ecosystem relationship between the time since ecosystem restoration, and the proportion of the reference carbon accumulated[9]. This relationship was estimated from a systematic meta-analysis, but represents a general pattern across blue carbon ecosystems, rather than being mangrove specific. There is no comparable global meta-analysis specific to mangroves, but there are case studies of accumulation of mangrove soil carbon stocks[10] and biomass[11] in foresting mangroves (Supplementary Fig. 7). Accumulation curves for the proportion of soil carbon[10] and biomass volume[11] present in afforesting mangroves were taken from two case studies from the published literature[11,15]. Biomass volume was used as a proxy of above- and belowground carbon[11,15]. In Sensitivity Analysis 4 we used these alternate a_t values to investigate the impacts of using mangrove-specific values. It was not possible to incorporate uncertainty in these values. All other parameters were maintained at the values used in the baseline study.

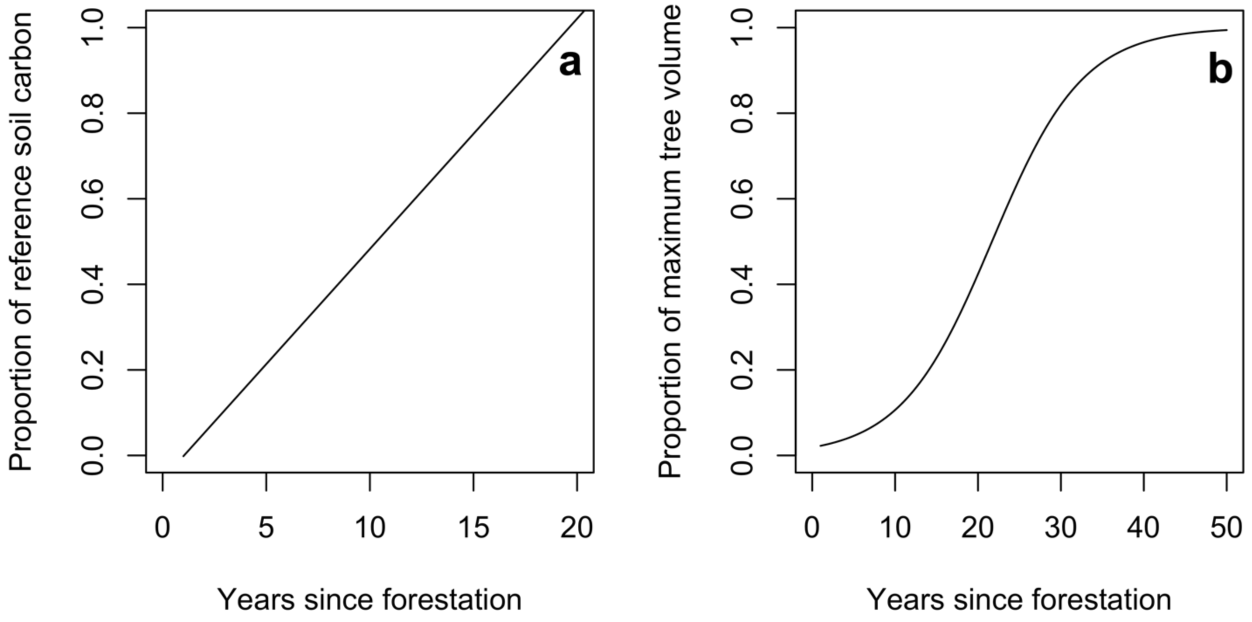

Supplementary Fig. 7. Temporal accumulation of (a) mangrove soil organic carbon stocks and (b) mangrove tree volume.

Sensitivity analysis results and discussion
Sensitivity analysis 1 gave estimates that differed the most from the baseline simulation (Supplementary Fig. 8), with a 35% lower median estimate. Sensitivity analysis 2 and 3 gave similar results to each other, with medians that were 27%

higher than the baseline. Sensitivity analysis 4 gave almost identical results to the baseline estimate (Supplementary Fig. 8). All of the median estimates given by the four sensitivity analyses were well within the 95% confidence intervals of the baseline estimate, although there is considerable uncertainty surrounding the baseline estimate.

Sensitivity analysis 1 reiterates the argument that past research has substantially over-estimated mangrove carbon stocks, by over-estimating mangrove soil organic carbon content through the use of an inappropriate conversion factor from loss-on-ignition[6]. For the purpose of the current study, we chose to continue with the previously-published global mangrove soil organic carbon datasets, as our method of estimating *OC* in order to make a correction factor should be regarded as indicative rather than robust. However, it is likely that the net losses in global mangrove carbon between 1996 and 2016 may be even lower than reported in our study. As future field measurements and meta-analyses begin to incorporate Ouyang and Lee's conversion factor, we may expect new datasets to become available that could be used to re-estimate global changes in mangrove carbon stock, following the framework we developed here.

Sensitivity analysis 2 and 3 gave higher estimates of the loss of mangrove carbon stocks, because they used overall mean r_t values rather than taking into account the amount of time since mangrove deforestation. The overall mean r_t values synthesised by the meta-analysis were high because most of the studies compared reference and deforested mangrove areas several years after the deforestation events[8]. The r_t values used in Sensitivity analyses 2 and 3 are therefore likely to represent the longer-term carbon stock losses that will eventually result from the mangrove deforestation observed between 1996 and 2016. The minor differences between the results of sensitivity analysis 2 and 3 suggest that it is not too critical to account for land-cover dependent r_t values, because these values do not differ greatly between replacement land cover types[8]. Sensitivity analysis 4 gave almost identical results to the baseline simulation, indicating that the simulation is not sensitive to the parameterisation of a_t value. This may partly be due to the broadly similar shape of the a_t curves used in the baseline simulation and sensitivity analysis 4. Perhaps more significantly, the relatively lower area of forestation means that this parameter has a relatively low weight in impacting the final outcomes for $Dr_t - Fa_t$.

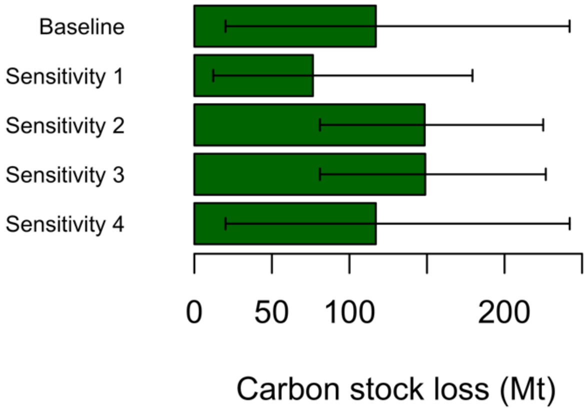

Supplementary Fig. 8. Sensitivity analysis of net loss in Southeast Asian mangrove carbon stock ($Dr_t - Fa_t$) between 1996 and 2016. Error bars indicate 95% bootstrap confidence intervals.

Supplementary Methods 2. Mangrove gain and loss classification errors
The GMW classification of mangrove forest has an error rate[2], leading to quantifiable uncertainty over the presence of mangroves at each location in 1996 and 2016. For each pixel of recorded mangrove forestation or deforestation, there is a probability that it is an erroneous or false positive example. For each pixel that is recorded as mangrove in both 1996 and 2016, or non-mangrove in both 1996 and 2016, there is again probability of error – a false negative case of gain or loss. Here we evaluate the potential for these errors to bias estimates of mangrove carbon stock loss and gain.

The false positive error-corrected area of mangrove forestation (G_p) can be estimated following the equation;

$$G_p = G_r \times M_c \times N_c$$

In which G_r is the area of mangrove forestation reported in the GMW dataset, M_c is the probability that a pixel reported in GMW as mangrove was correctly reported as mangrove, and N_c is the probability that a pixel reported as non-mangrove was correctly reported thus.

The false negative error-corrected area of mangrove forestation (G_n) can be estimated as;

$$G_n = (L_r \times M_m \times N_m) + (S_r \times M_m \times M_c) + (U_r \times N_c \times N_m)$$

In which L_r is the area of mangrove deforestation, S_r is the area of mangrove reported in both 1996 and 2016, and U_r is the area of non-mangrove reported, in both 1996 and 2016 in the GMW dataset. The M_m and N_m terms refer to the probabilities that mangrove and non-mangrove pixels reported by GMW were misclassified.

The total error-corrected area of mangrove forestation (G_t) can be summarised as;

$$G_t = G_p + G_n$$

Similarly, the false positive error-corrected area of mangrove deforestation (D_p) can be estimated following the equation;

$$D_p = L_r \times M_c \times N_c$$

The false negative error-corrected area of mangrove deforestation (L_n) can be estimated as;

$$D_n = (G_r \times M_m \times N_m) + (S_r \times M_m \times M_c) + (U_r \times N_c \times N_m)$$

And the total error-corrected area of mangrove forestation (D_t) can be summarised as;

$$D_t = D_p + D_n$$

The probabilities of mangrove and non-mangrove classification error (N_c, N_m, M_m, and M_c) can be taken from the GMW classification error matrix[2]. As error matrices for all years in GMW are not available, we assume that they all follow the same error rates as 2010, which is the best documented year[2]. The areal extent of G_r , L_r, and S_r can be calculated by cross-referencing the 1996 and 2016 layers of GMW mangrove extent[2–4]. However, to our knowledge, the areal extent of non-mangrove classified by GMW (U_r) was not reported in the associated publications and documentation[2–4], making it challenging to robustly assess the total error-corrected area of mangrove forestation and deforestation. For the purpose of evaluating potential bias introduced by false positive and negative-error correction, we take a conservative estimate of the area of non-mangrove under the GMW study, and assume that the area of U_r is equal to S_r (Supplementary Table 6).

Supplementary Table 6. Estimation of false positive- and false negative- corrected areas of mangrove forestation and deforestation area, using the assumption that U_r is equal to S_r.

Parameter	Description	Value
G_r	GMW-reported area of forestation	2,379.3
L_r	GMW-reported area of deforestation	8,538.6
S_r	GMW-reported area of mangrove present in both 1996 and 2016	136,707.2
U_r	GMW-reported area of non-mangrove present in both 1996 and 2016	136,707.2
M_m	Probability that reported mangrove is misclassified as non-mangrove	0.025
M_c	Probability that reported mangrove is classified correctly	0.975
N_m	Probability that reported non-mangrove is misclassified as mangrove	0.033
N_c	Probability that reported mangrove is classified correctly	0.967
G_p	False positive error-corrected estimate of mangrove forestation	2,243.3
G_n	False negative error-corrected estimate of mangrove forestation	7,701.7
D_p	False positive error-corrected estimate of mangrove deforestation	8,050.4
D_n	False negative error-corrected estimate of mangrove deforestation	7,696.7

The false negative error-corrected estimates of mangrove forestation and deforestation area were very similar, with the ratio of G_n: D_n almost 1:1. This is due to the similarity of the equations used to generate these estimates, as both incorporate the ($S_r \times M_m \times M_c$) and ($U_r \times N_c \times N_m$) components. The area of mangrove and non-mangrove that does not change between 1996 and 2016 is greatly larger than either the area of gain or loss, so this part of the calculation largely determines the estimate. Conversely, the ratio of G_p: D_p is more than 1:3, indicating that the false positive error rate contributes more to overestimating deforestation than forestation.

In the study, we included uncertainty in mangrove forestation and deforestation due to the false positive error rate in the bootstrap simulation, because of the potential bias that would occur if this were not accounted for. We did not include false negative error corrections due to the lack of U_r data available to estimate these values, and the negligible bias towards either forestation or deforestation area caused by omitting these corrections.

Supplementary Methods 3. Simulation of y-values from new x-values in linear model

We modelled the proportion of mangrove carbon remaining as a function of the length of time since deforestation, and mangrove carbon accumulated as a function of the time since forestation, by simulating linear models to predict the relevant *y* values for a given *x* value, while taking into account the errors present in the model. This was implemented by a modification of the simulate function in the R statistical computing software[14]. The following code was developed by user Josh O'Brien in response to a query on Stack Overflow: https://stackoverflow.com/questions/14967813/is-there-a-function-or-package-which-will-simulate-predictions-for-an-object-ret [accessed 17th May 2020].

```
simulateX <- function(object, nsim=1, seed=NULL, X, ...) {

  X2<- predict(object, data.frame(years=X), type="response")

  object$fitted.values <- X2
  simulate(object=object, nsim=nsim, seed=seed, ...)
}
```

Supplementary Methods 4. Meta-analysis of blue carbon accumulation following restoration

The whole-ecosystem carbon accumulation curve for foresting mangroves was estimated using data taken from a meta-analysis of blue carbon ecosystem restoration[9]. We extracted only data points from studies that compared carbon indicator values measured in restored ecosystems against a natural reference ecosystem[9]. We estimated the log carbon response ratio (*lnrr*) following restoration as a proportion of the reference ecosystem carbon, defined for studies that used a before/after or control/impact study design as;

$$lnrr = log(T/C)$$

In which T is the treatment carbon indicator value and C is the control carbon indicator value. For studies that used a before/after/control/response study design, we defined the log carbon response ratio as;

$$lnrr = log((T_a/C_a) - (T_p/C_p))$$

In which T_a is the treatment carbon indicator after treatment, C_a is the control carbon indicator value after treatment, T_p is the treatment carbon indicator before treatment, and C_p is the control carbon indicator value before treatment.

We then modelled the resulting log carbon response ratios using a log-linear regression model as a function of the year since restoration[9]. The regression model showed a statistically significant effect of log(year) on the log carbon response ratio (Supplementary Table 7; Supplementary Fig. 3c).

Supplementary Table 7. Log-linear relationship between years since restoration and log carbon response ratio. Adjusted R^2 for the model is 0.12.

Coefficient	Estimate	Standard error	t	p
Intercept	-0.866	0.121	-7.2	< 0.001
log(years)	0.249	0.062	4.0	< 0.001

Supplementary references

1. Hutchison, J., Manica, A., Swetnam, R., Balmford, A. & Spalding, M. Predicting global patterns in mangrove forest biomass. *Conserv. Lett.* **7**, 233–240 (2014).
2. Bunting, P. *et al.* The global mangrove watch - A new 2010 global baseline of mangrove extent. *Remote Sens.* **10**, (2018).
3. Thomas, N. *et al.* Mapping mangrove extent and change: A globally applicable approach. *Remote Sens.* **10**, 1–20 (2018).
4. Thomas, N. *et al.* Distribution and drivers of global mangrove forest change , 1996 – 2010. *PLoS One* e0179302 (2017).
5. Sanderman, J. *et al.* A global map of mangrove forest soil carbon at 30 m spatial resolution. *Environ. Res. Lett.* **13**, (2018).
6. Ouyang, X. & Lee, S. Y. Improved estimates on global carbon stock and carbon pools in tidal wetlands. *Nat. Commun.* **11**, 1–7 (2020).
7. Global Mangrove Watch. Global Mangrove Watch 1996 - 2016. *UNEP-WCMC Data Viewer* (2020). Available online: https://data.unep-wcmc.org/ Accessed 28th July 2020.
8. Sasmito, S. D. *et al.* Effect of land-use and land-cover change on mangrove blue carbon: A systematic review. *Glob. Chang. Biol.* gcb.14774 (2019). doi:10.1111/gcb.14774
9. O'Connor, J. J., Fest, B. J., Sievers, M. & Swearer, S. E. Impacts of land management practices on blue carbon stocks and greenhouse gas fluxes in coastal ecosystems—A meta-analysis. *Glob. Chang. Biol.* **26**, 1354–1366 (2020).
10. Osland, M. J. *et al.* Ecosystem Development After Mangrove Wetland Creation: Plant-Soil Change Across a 20-Year Chronosequence. *Ecosystems* **15**, 848–866 (2012).
11. Sillanpää, M., Vantellingen, J. & Friess, D. A. Vegetation regeneration in a sustainably harvested mangrove forest in West Papua, Indonesia. *For. Ecol. Manage.* **390**, 137–146 (2017).
12. Richards, D. R. & Friess, D. A. Rates and drivers of mangrove deforestation in Southeast Asia, 2000–2012. *Proc. Natl. Acad. Sci.* **113**, 344–349 (2016).
13. Goldberg, L., Lagomasino, D., Thomas, N. & Fatoyinbo, T. Global declines in human-driven mangrove loss. *Glob. Chang. Biol.* doi: 10.1111/gcb.15275 (2020). doi:10.1111/gcb.15275
14. R Core Team. R: A Language and Environment for Statistical Computing. *R: A Language and Environment for Statistical Computing* (2017). Available online: www.R-project.org/ Accessed 28th July 2020.
15. Rovai, A. S. *et al.* Scaling mangrove aboveground biomass from site-level to continental-scale. *Glob. Ecol. Biogeogr.* **25**, 286–298 (2016).
